# Intrafollicular barriers and cellular interactions during ovarian follicle development

**DOI:** 10.21451/1984-3143-AR2019-0051

**Published:** 2019-10-23

**Authors:** Gabriella Mamede Andrade, Maite del Collado, Flávio Vieira Meirelles, Juliano Coelho da Silveira, Felipe Perecin

**Affiliations:** 1 Faculty of Animal Sciences and Food Engineering, Department of Veterinary Medicine, University of São Paulo, Pirassununga, São Paulo, Brazil.

**Keywords:** cellular communication, extracellular vesicles, granulosa cells, oocyte, ovarian follicle, transzonal projections

## Abstract

Follicles are composed of different interdependent cell types including oocytes, cumulus, granulosa, and theca cells. Follicular cells and oocytes exchange signaling molecules from the beginning of the development of the primordial follicles until the moment of ovulation. The follicular structure transforms during folliculogenesis; barriers form between the germ and the somatic follicular cells, and between the somatic follicular cells. As such, communication systems need to adapt to maintain the exchange of signaling molecules. Two critical barriers are established at different stages of development: the zona pellucida, separating the oocyte and the cumulus cells limiting the communication through specific connections, and the antrum, separating subpopulations of follicular cells. In both situations, communication is maintained either by the development of specialized connections as transzonal projections or by paracrine signaling and trafficking of extracellular vesicles through the follicular fluid. The bidirectional communication between the oocytes and the follicle cells is vital for driving folliculogenesis and oogenesis. These communication systems are associated with essential functions related to follicular development, oocyte competence, and embryonic quality. Here, we discuss the formation of the zona pellucida and antrum during folliculogenesis, and their importance in follicle and oocyte development. Moreover, this review discusses the current knowledge on the cellular mechanisms such as the movement of molecules via transzonal projections, and the exchange of extracellular vesicles by follicular cells to overcome these barriers to support female gamete development. Finally, we highlight the undiscovered aspects related to intrafollicular communication among the germ and somatic cells, and between the somatic follicular cells and give our perspective on manipulating the above-mentioned cellular communication to improve reproductive technologies.

## **Introduction: Follicle developmen**t

The ovarian follicle development starts long before birth during the intra-uterine period (Russe, 1983). The primordial germ cells migrate to the genital ridge, colonize, and proliferate. After this highly proliferative period, a human female fetus has approximately 6-7 million germ cells around the 20^th^ week of gestation, however a vast majority of these germ cells are lost and approximately 1 to 2 million oocytes remain viable at birth (Motta *et al*., 1997; Sun *et al*., 2017). In bovines, the maximum number of germ cells is around 2.5 million at about the 15^th^ week of gestation (Erickson, 1966) and thirteen days after birth bovine germ cells number decrease approximately to 68 thousand. This dramatic loss of germ cells close after birth occurs in most female mammals (Paulini *et al*., 2014).

Once mitotic proliferation stops, these germ cells arrest at meiotic prophase I to form the germ cell nests (Buehr, 1997; Tilly, 2001; Sun *et al*., 2017). Close to birth, breakdown of the germ cell nests occurs with the formation of the primordial follicle. Two cell types characterize this primordial follicle: a primary oocyte surrounded by a single layer of pre-granulosa cells (Fortune, 1994; BrawTal and Yossefi, 1997; Eppig, 2001). The primordial follicle population in the ovary serves as a reservoir for developing follicles and oocytes throughout the female reproductive life (Zuckerman, 1951; Kerr *et al*., 2013). After puberty, groups of primordial follicles are periodically recruited to initiate folliculogenesis.

Although the precise mechanisms that regulate germline nest breakdown and primordial follicle formation are mostly unknown (Wang *et al*., 2017), several growth factors and hormones play essential roles in primordial follicle formation (Pepling, 2012), for example estradiol-17β (E2) and members of the transforming growth factor beta (TGF-β) superfamily (Knight and Glister, 2006; Wang and Roy, 2007; Chakraborty and Roy, 2017). The TGF-β family members are secreted by the oocyte and include bone morphogenetic protein 15 (BMP15) and growth differentiation factor 9 (GDF9), which act via autocrine and paracrine mechanisms, regulating follicle growth and differentiation, as well as granulosa and thecal cell function during follicular development (Dong *et al*., 1996; Eppig *et al*., 1997; Gilchrist *et al*., 2004; Sanfins *et al*., 2018). By secreting these members of TGF-β family the oocyte is the main responsible for activating primordial follicles (Eppig, 2001).

Ovarian follicle development is a continuous process that has two different phases: the preantral and antral. The first phase, preantral, is gonadotropin-independent and relies on local growth factors. As folliculogenesis progresses, the follicle becomes gonadotropin-responsive and develops until secondary follicles. The second phase, antral, is gonadotropin-dependent and is characterized by the presence of the tertiary follicles, which has the presence the antrum, a cavity filled with follicular fluid (Dvořák and Tesařík, 1980; Erickson and Shumichi, 2001). This entire process of growth and differentiation of the follicle is accompanied by the oocyte growth and acquisition of competence (El-Hayek and Clarke, 2015; Monniaux, 2016).

Stimulation by the locally secreted factors activates the primordial follicles initiating the preantral growth phase for development into a primary follicle. Factors responsible for primary follicle development are not fully known; however, it is known that granulosa cell-derived anti-Mullerian hormone and activins participate in the regulation of this process (reviewed by Matzuk *et al*., 2002). The primary follicles are characterized by the presence of an oocyte covered with a single layer of cuboidal granulosa cells. As the oocyte grows, the granulosa cells proliferate to envelop the surface of the expanding oocyte (vandenHurk *et al*., 1997).

**Figure 1:**
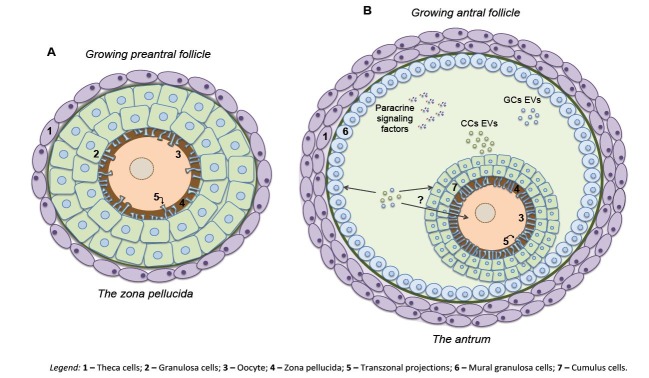
*Physical barriers to cell-to-cell communication in the ovarian follicle are established during folliculogenesis.* During preantral growth, zona pellucida, the first significant barrier between the oocyte and follicular cells, is formed. This barrier between the germ and somatic cells results from the deposition of glycoproteins by the oocyte. For continuous maintenance of a cytoplasmic bridge between germ and somatic cells, the oocyte stimulates the granulosa cells to generate specialized cytoplasmic filaments connecting both cells – the transzonal projections **(A)**. In antral growing follicles, a second significant barrier among follicular cells is formed – the antrum. Bilateral communication is maintained by paracrine signaling and extracellular vesicle traffic. Paracrine signaling of oocyte-secreted factors and transactivation of the EGF receptor by LH signaling drives follicle development and ovulation. Extracellular vesicles are secreted into the follicular fluid and are taken up by different cells types by a cargo delivery mechanism. The direct transfer of EVs-cargo from the follicular fluid to the oocyte remains elusive **(B)**. CCs – cumulus cells; EVs – extracellular vesicles; GCs – granulosa cells; Theca – theca cells.

Continuous granulosa cell proliferation results in multiple layers of cells surrounding the oocyte and the follicles are referred to as secondary follicles. At this stage, the formation of the theca cell layer starts, separated from the granulosa by a basement membrane (BrawTal and Yossefi, 1997). At the same time, oocytes undergo alterations as the formation of cortical granules in the cytoplasm (Fair *et al*., 1997) and the beginning of mRNA synthesis (McLaughlin *et al*., 2010). At this stage, the formation of the zona pellucida (ZP) around the oocyte starts, to form the first significant barrier between the oocyte and the somatic granulosa cells (BrawTal and Yossefi, 1997; Clarke, 2018) (Fig. 1A).

As the secondary follicle develops, more layers of granulosa cells form, and an antral cavity filled with follicular fluid develops between them. With the initiation of the antral phase of follicular growth, the follicle is now a tertiary follicle. During the transition of the secondary to tertiary follicle the second significant barrier between follicular cells is formed (Fig. 1B). Indeed, the antrum induces the differentiation of granulosa subpopulations, the original granulosa cells present in the outer wall of the follicle and that specialized granulosa cells, now cumulus cells, that directly surround the oocyte during further development. Mural granulosa cells and cumulus cells became exposed to opposing gradients of follicle-stimulated hormone (FSH) and oocyte-secreted factors (OSF) (Fortune, 1994; Eppig, 2001; Wigglesworth *et al*., 2015). In this phase of intense follicle growth, the oocyte slows down or even stops its growth, while stromal cells form two layers of cells external to the basement membrane, the internal and external theca cell layers (Fair *et al*., 1997; Hyttel *et al*., 1997; Guo *et al*., 2016).

In bovine, from the beginning of antral phase until a diameter of approximately 8 mm, follicle growth is stimulated by FSH secreted by the pituitary gland. Follicles develops by the rapid proliferation of granulosa and theca cells that contribute to the further enlargement of the antrum and the follicle itself. From a diameter of 8 mm onwards, the follicle develops mainly by the trophic stimulation of LH, and eventually, after the LH surge, will be termed preovulatory follicles (Eppig *et al*., 1997).

At the end of its growth, the dominant follicle reaches a plateau phase of non-exponential growth with fewer cell divisions and slower diameter increase (Girard *et al*., 2015). Following the preovulatory gonadotropin surge, follicular cells initiate morphological, endocrine, and biochemical changes associated with luteinization process (Smith *et al*., 1994; Revelli *et al*., 2009). In monovulatory species, only one follicle continues its growth to become an ovulatory follicle, while the remaining antral follicles regress and undergo atresia (Hennet and Combelles, 2012).

For the follicle formation and its steady growth during the whole folliculogenesis process, the bidirectional communication within the follicle environment is essential for the complete development of the follicle as well as the oocyte. The crosstalk between the oocyte and somatic follicular cells and between the somatic follicular cells occurs through the interactions mediated by paracrine signaling factors, by gap junctions and, as recently described, by extracellular vesicles. The paracrine signaling occurs through the secretion of factors from the oocyte or from the somatic cells. The gap junctions are structures formed by connexins that allow the transport of molecules of low molecular weight (<1 kDa) as ions, metabolites and amino acids between granulosa cells and cumulus cells, and between cumulus and oocyte cells. These junctions connect neighbor follicular cells or germ and somatic cell, at the bulk end of transzonal projections. The extracellular vesicles consist in a communication system mediated by vesicles secreted by cells. These vesicles, may have proteins, miRNAs and mRNAs as cargo, and are secrete and uptake by follicular cells (reviewed by Del Collado *et al*., 2018).

**Figure 2:**
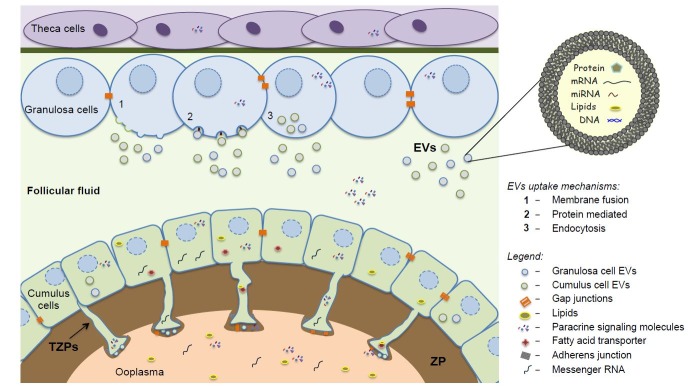
*Bidirectional communication within the ovarian follicle microenvironment.* The bidirectional crosstalk between cells that compose the follicle is associated with follicular development and acquisition of oocyte competence. Cellular crosstalk between germ-somatic cells and between somatic cells is mediated by the secretion of paracrine factors, by the communication through transzonal projections (TZPs) via gap junctions, and via extracellular vesicles (EVs) trafficking from the bulk end of TZPs to the oocyte, or trafficking into the follicular fluid. TZPs are specialized cytoplasmic projections that extend across zona pellucida (ZP) and allow the exchange of small molecules such as sugars, pyruvate, amino acids, and nucleotides, and large molecules such as mRNAs, lipids, and small organelles. Follicular fluid EVs are lipid bilayer vesicles loaded with proteins, mRNAs, microRNAs, lipids, and DNA and are taken up through endocytosis, protein recognition, and membrane fusion by distinct cell types within the ovarian follicle allowing communication with distant cells.

Hence, there are two physical barriers existing in the follicular environment, the ZP and the antrum. In both cases, cellular communication mechanism overcomes these barriers to maintain the exchange of messages via transzonal projections (TZPs) and extracellular vesicles (EVs; Fig. 2). These barriers, the communication mechanisms within, and the importance of such communication for the follicle and oocyte development are discussed in the following part of the review.

## The first barrier

### ZP formation and the germ-soma barrier

The ZP is a relatively thick extracellular coat that surrounds all mammalian oocytes. It is formed in the preantral phase of follicular development during the formation of secondary follicles, when the oocytes are arrested in the late diplotene stage and undergoing active growth (Wassarman and Litscher, 2012). This porous extracellular coat is formed by three or four glycoproteins depending on the species (Bleil and Wassarman, 1980; reviewed by Gupta, 2015). The ZP has essential functions during oogenesis, fertilization, and pre-implantation development (Wassarman and Litscher, 2012). For example, during oocyte development, the ZP integrity is important to maintain the communication between the oocyte and the cumulus cells (Wang *et al*., 2019). During fertilization the ZP play fundamental roles, as the block of non-specific fertilization, the block of polyspermia (Wassarman, 1999; Florman and Ducibella, 2006). And during early embryo development the ZP permit that cleavage stage embryos move freely along the oviduct and protect the growing embryo until implantation (Gupta *et al*., 2015) so that the ZP presence is necessary for normal early development in uterus (Modliński, 1970).

As an oocyte increases in diameter, its ZP increases in thickness (Wassarman and Litscher, 2013) and separate the oocytes and the surrounding cumulus cells. Depending on the species, the ZP ranges in thickness from less than 1 mm to more than 25 mm (Keefe *et al*., 1997). Despite this physical separation, the oocytes and cumulus cells maintain contact by the formation of TZPs (Albertini *et al*., 2001; Eppig, 2018). TZPs mainly originate from the cumulus cells in the layer immediately adjacent to the oocyte, but they are also shown to arise from cumulus cells positioned in oocyte more distant layers (Jaffe and Egbert, 2017). Since the number of TZPs present in the ZP of a developing oocyte is quite high, it is likely that each somatic cell surrounding the oocyte emits multiple projections towards the oocyte. Also, some projections extend from a single point of origin at the granulosa cell membrane and subsequently dividing into several TZPs towards the ooplasm (El-Hayek *et al*., 2018).

There are two hypotheses for the formation of TZPs: by “stretching” or “pushing.” In the “stretching” model, the adhesion sites between the oocyte and granulosa cells are already present before ZP formation and remain during the ZP deposition process, to become stretched cytoplasmic filaments called TZPs. The second hypothesis, known as “pushing,” proposes that the TZPs are elaborated from the granulosa cells after the deposition of the ZP and grow towards the oocyte where they establish contact with their plasma membrane (Clarke, 2017).

The growing oocyte induces somatic cells to generate the TZPs (El-Hayek *et al*., 2018). Factors secreted by the oocyte, such as GDF9 and FSH, correlate with the development of TZPs. Recent studies show that GDF9 produced by the oocyte acts via the SMAD signaling pathway to stimulate neighboring granulosa cells to generate TZP structures (El-Hayek *et al*., 2018). Besides that, recent functional studies verified that GDF9 maintains stable mRNAs that encode TZPs structural components (El-Hayek *et al*., 2018) and the absence of oocyte GDF9 leads to morphologically abnormal TZPs in mice (Dong *et al*., 1996; Carabatsos *et al*., 1998). FSH induces the retraction of TZPs, but the specific pathways by which this happens are still unclear (Combelles *et al*., 2004).

### Oocyte and soma interactions through TZPs

The TZPs form concomitantly with the ZP and are specialized filopodia characterized as communication channels of approximately 2 um in diameter without the fusion of membranes (Macaulay *et al*., 2014). These channels originate from the cytoplasmic filaments of actin or tubulin, and their function depends on the composition. TZPs formed by tubulin filaments are related to cell adhesion while actin TZPs are involved in cell communication, and the latter are prevalent in oocyte ZP (Li and Albertini, 2013).

These projections allow communication between the oocyte and somatic cells. As the TZPs are free-ended structures, the exchange of small molecules occurs at the bulk end of the projections by gap junctions and intermediate junctions (zonula adherens-like junctions) that keep the cytoplasmic membranes of both cells in close contact (Hyttel *et al*., 1997; Albertini and Barrett, 2004). Additionally, at the bulk end of TZPs, a cleft is formed between the plasma membrane of the TZP and the oolemma. Extracellular vesicles were identified at this cleft (Macaulay *et al*., 2014) and are involved in potential mechanisms by which cargo transfer occurs from somatic cells to oocyte.

Some molecules, such as mRNAs, lipids, pyruvate and cGMP, are shown to be transported through TZPs, suggesting the importance of these communication mechanisms between the oocyte and the surrounding cumulus cells. TZPs have distinct roles, for example: i) in mRNA accumulation, as evidenced by passage of polyadenylated transcripts (Macaulay *et al*., 2014; Macaulay *et al*., 2016), ii) in metabolic and nutritional cooperation, due to continuous exchange of small molecule ions, cyclic nucleotides, and amino acids (Thomas *et al*., 2004; Lodde *et al*., 2013), iii) and providing energy substrates such as pyruvate, lactate (Scantland *et al*., 2014), and other metabolites. Recent results show that TZPs also have a role in lipid transport from cumulus cells to oocyte. The TZPs lipid transport was proved by co-localization of fatty acid binding protein 3 (FABP3), a protein responsible for carrying lipids, with TZPs within zona pellucida and by the increase of oocyte lipid droplets dependent on the presence of TZPs, indicating that TZPs might be involved in the oocyte lipid accumulation during maturation (del Collado *et al*., 2017).

The communication through TZPs have fundamental role in the oocyte meiosis control and oocyte maturation, since the transport of essential molecules as cAMP, is mediated by TZPs from somatic cells to the oocyte (Eppig *et al*., 2005; Gilchrist *et al*., 2016). *In vitro* studies with bovine cumulus-oocyte complex revealed that the communication is maintained until the resumption of meiosis and onset of detachment within 9 h of maturation, and gradually decreases up to 22 h, when it eventually comes to a stop (Macaulay *et al*., 2014). A recent study pointed out that inclusion of the pre-IVM phase with a combination of cAMP modulators, resulted in maintenance of the density of TZPs after 20 h of *in vitro* maturation, resulting in improvement in the cumulus-oocyte communication leading to enhanced oocyte developmental competence (Soto-Heras *et al*., 2019). In aging females, the ability of somatic cells to respond to oocyte signals is reduced, resulting in lower formation of TZPs. Consequently, the reduced oocyte-somatic cell communication is the presumed cause for the reduced fertility in aged females (El-Hayek *et al*., 2018).

Interestingly, most of the time the TZPs are not in contact with the oocyte, they subdivide and form gap junctions between each other (Baena and Terasaki, 2019), a sign that these projections also have other essential functions such as communication between somatic cells. The TZPs are involved in essential processes for oocyte and consequently, embryo development. Studies investigating the transport mechanisms present in TZPs and how the *in vitro* environment influences these projections are still ongoing. This knowledge will probably be helpful to prevent lipid accumulation, aging consequences, and to improve *in vitro* oocyte maturation, with broad implications for animal and human assisted reproduction technologies.

## The second barrier

### Antrum formation and the cumulus-granulosa barrier

The antral follicles are characterized by the formation of a cavity filled with the follicular fluid. The follicular fluid originates from two sources, the bloodstream of thecal capillaries present in the ovary cortical region, and the components secreted by follicular cell layers, especially the granulosa cells and the fluid production, which intensifies with the enlargement of the follicles (Rodgers and Irving-Rodgers, 2010; Hennet and Combelles, 2012). The main hypothesis on follicular fluid formation suggests that an osmotic gradient is generated by granulosa cells production of hyaluronan and the chondroitin sulfate proteoglycan versican. This gradient generates influx of fluid derived from the thecal vasculature (Rodgers and Irving-Rodgers, 2010).

The follicular fluid contains a complex mixture of ions, proteins, metabolites, hormones, lipids, energy substrates, and reactive oxygen species (Leroy *et al*., 2004; Meeker *et al*., 2009; Ambekar *et al*., 2013). It serves as a source of regulatory molecules, such as gonadotrophins, steroids, growth factors, enzymes, proteoglycans, and lipoproteins (Revelli *et al*., 2009). This diverse array of molecules suggest that the follicular fluid is more than a reservoir and also supports intense metabolic activity, with substantial impact on follicular cells (Freitas *et al*., 2017) and oocyte. Roles of the follicular fluid were already reported, such as the participation in oocyte’s acquisition of developmental competence (Fayezi *et al*., 2014; O’Gorman *et al*., 2013; Wallace *et al*., 2012) and in oocyte meiosis (Byskov *et al*., 1995; Mendoza *et al*., 2002). For example, hormone level in the follicular fluid, such as FSH (Suchanek *et al*., 1988), hCG (Ellsworth *et al*., 1984; Enien *et al*., 1998) and LH (Cha *et al*., 1986) have been reported to promote oocyte maturation and to increase chances of fertilization. The gonadotropins induce granulosa cells to secret hyaluronic acid (Mendoza *et al*., 2002) affecting oocyte development; they also act synergistically with estradiol (E2) enhancing cytoplasmatic maturation and controlling oocyte meiosis via cAMP secretion (Mendoza *et al*., 2002; Revelli *et al*., 2009). Another example is the fatty acids found in the follicular fluid that are incorporated by the oocyte and that have influence on oocyte maturation and quality. Excess of fatty acids were reported to negatively impact fertility outcomes (Shaaker *et al*., 2012).

The signaling mechanism for the formation of the antrum is not well understood; however, it was shown that FSH and type 1 insulin-like epidermal growth factors promote the formation of the antrum in cultured follicles *in vitro* (Gutierrez *et al*., 2000; Hillier, 2009). The growth of the antral follicles in bovines occurs in two distinct phases. The first is the slow phase where the follicles take approximately 30 days to advance from 0.3 mm in diameter to the stage of small antral follicles, which are about 3 mm in diameter. In this period of follicular growth, the oocyte reaches its final growth, approximately 110 µm in diameter, which relates to the acquisition of competence for development (Fair *et al*., 1997; Rodriguez and Farin, 2004). The second phase is the active phase when small follicles, approximately 3 mm in diameter, take from five to seven days to become dominant follicles, more than 8 mm in diameter. This phase is followed by a variable period of dominance, culminating in the development of the preovulatory follicle and ovulation (Bleach *et al*., 2001; Mihm and Bleach, 2003).

Given that antrum formation separates follicular cells and gametes, the need to maintain the communication between these cells is accomplished mainly through paracrine signaling. An example of paracrine signaling is the oocyte-secreted factors (GDF9 and BMP15) that interact with molecules such as FSH, IGF1 and androgens to promote mural granulosa proliferation and cumulus cells differentiation (Gilchrist *et al*., 2004).

Other examples of critical paracrine factors are found in preovulatory follicle. The signaling cascade triggered by the pre-ovulatory LH peak propagate through the ovulatory follicle via paracrine factors and stimulates the release of epidermal growth factor (EGF) ligands from the mural granulosa cells, that move across the follicular fluid to reach the cumulus cells. In these target cells induce changes in gene expression that will decrease the cGMP concentration in the cumulus cells and consequently in the oocyte, resulting in cumulus cells expansion and meiosis resumption (Conti *et al*., 2012). The OSF also play vital roles regulating extracellular matrix stability, leading to ovulation (Gilchrist *et al*., 2004). Thus, this cascade is essential for the induction of gene expression required for follicle rupture, oocyte maturation and ovulation.

### A novel communication system in the antrum

While paracrine signaling communication in follicular fluid have been described for many years, a novel communication mechanism, mediated by extracellular vesicles, has recently been described. Extracellular vesicles are phospholipid bilayer vesicles that transport biomolecules such as proteins, microRNAs, mRNAs, DNA, and lipids (Taylor and Gercel-Taylor, 2013; Di Pietro, 2016; Ávila *et al*., 2019). Their content varies and reflects the cell of origin (Akers *et al*., 2013). It was shown that the secretory cells actively select the number and the cargo of EVs depending on specific physiological and environmental conditions, such as diseases, nutritional status and stress (van Niel *et al*., 2018).

EVs are classified into microvesicles (MVs) and exosomes (Exos) according to their characteristics such as size, shape, membrane proteins, structural lipids, and their origin. The MVs are big with a diameter ranging from 500-1000 nm, have an irregular shape, and originate from the rupture of the cellular plasma membrane, which makes MVs a more heterogeneous population. On the other hand, the Exos have an approximate diameter of 50-100 nm (Crescitelli *et al*., 2013) and appear in electron microscopy as a cup-shaped form, depending on the preparation method. Importantly, EVs originate from the late endosomes, also called multivesicular bodies and are released into the extracellular space by fusion of the multivesicular body membrane with the plasma cell membrane (Taylor and Gercel-Taylor, 2013).

There are three modes of interaction between the EV and their target cells; i) the first is through direct interaction between membrane proteins of the EV with receptors on the target cell membrane, ii) The second by membrane cleavage of the EV proteins by proteases present in the extracellular space, and the release of products which act on the receptors of the target cell, and iii) the third by direct fusion of the EV membranes with the cell membrane, releasing the EV content in the cell and incorporating proteins and receptors into the cell membrane (Mathivanan *et al*., 2010).

EVs are present in several body fluids and were first described in the follicular fluid a few years ago (da Silveira *et al*., 2012). Follicular cells secrete these vesicles into the follicular environment (Andrade *et al*., 2017a) and the EVs are taken up by the granulosa and cumulus cells, in *in vivo* and *in vitro* systems (da Silveira *et al*., 2012; Di Pietro, 2016). Additionally, the oocyte surrounding cumulus cells could provide an entry point to deliver to oocyte the molecules that cannot pass through gap junctions, such as RNAs, miRNAs, proteins and lipids (Macaulay *et al*., 2014; Macaulay *et al*., 2016). Interestingly, *in vitro* studies showed that follicular fluid EVs alter transcript levels in oocytes (Dalanezi *et al*., 2017) and enhance oocyte competence to develop until the blastocyst stage (da Silveira *et al*., 2017).

The follicular fluid undergoes dynamic changes over late stages of folliculogenesis and its EVs content modify as consequence. As an example, the follicular fluid EVs from different size follicles have distinct concentrations and miRNA content (Navakanitworakul *et al*., 2016), and it has been described that they can differentially stimulate granulosa cells proliferation *in vitro* (Hung *et al*., 2017). Additionally, female age (Diez-Fraile *et al*., 2014; da Silveira *et al*., 2015a), and endocrine environment (da Silveira *et al*., 2015a) are important factors that can alter EVs content. Regarding female age, the miRNAs content of EVs from follicular fluid varies according to age. In old mares compare to young, a group of highly expressed miRNAs negatively modulates TGF-β, resulting in compromised maturation of oocytes (Da Silveira *et al*., 2015b). Also, the miR-23a, highly expressed in old mares (Da Silveira *et al*., 2015b), is correlated with human granulosa cells apoptosis pathway by inhibition of X-linked inhibitor of apoptosis protein (XIAP) and an increase in caspase 3 protein levels (Yang *et al*., 2012; Mobarak *et al*., 2019).

The bidirectional communication through EVs is associated with follicular development, oocyte growth, and quality, in humans and domestic animals (da Silveira *et al*., 2012; Sang *et al*., 2013; Sohel *et al*., 2013; Hung *et al*., 2015). During the maturation process, the EVs induce cumulus cell expansion and alter expression of genes related to the expansion process, when used as a supplement on cumulus-oocyte complexes (COCs) maturation medium (Hung *et al*., 2015). In another study, EVs stimulated granulosa cell proliferation by modulating Src, Pi3K/Akt and mitogen-activated protein kinase (MAPK) pathways, and interestingly, the EVs from small follicles were preferentially taken up by granulosa cells (Hung *et al*., 2017). Further, using EVs as supplements for embryo maturation in culture media, partially altered genes related to metabolism and development as well as miRNA and global DNA methylation and hydroxymethylation of bovine embryos produced *in vitro* (da Silveira *et al*., 2017). Another study observed a positive effect of follicular fluid EVs during *in vitro* maturation; they protected COCs from the harmful effects of heat shock stress (Rodrigues *et al*., 2019).

Although EVs carry different molecules, many studies show that the effect of EVs on cells are related to miRNAs and their regulatory effects, mainly because they are very stable and show resistance to degradation. The miRNAs in the follicular fluid EVs were associated with fertilization and embryo quality (Machtinger *et al*., 2017). Some studies, in humans and animals, have shown the role of miRNAs present in the follicular fluid, regulating follicular growth and development, cellular signaling, oocyte meiosis, and ovarian function (Martinez *et al*., 2018). EVs miRNAs modulate such important reproduction processes by the regulation of pathways as insulin, epidermal growth factor receptor (ErbB), MAPK, Wnt signaling, TGF-β and PI3K-Akt signaling among others (da Silveira *et al*., 2012; Santonocito *et al*., 2014; Andrade *et al*., 2017b). Due to the importance of cellular communication in this environment and the immense potential of EVs, efforts are dedicated to better understand its functions and importance during oocyte maturation and early embryo development *in vitro*.

## Conclusions and perspectives

Normal oocyte development depends on a finely regulated, constant, and reciprocal cell-to-cell communication between the follicle components. The crosstalk may occur by paracrine signaling and exchange of small molecules via gap-junctions; these are well-studied mechanisms. However, novel mechanisms of communication in the follicle microenvironment have been recently identified. These mechanisms allow the exchange of large molecules, such as nucleic acids, proteins, and lipids between follicular compartments, and are mediated by the trafficking of vesicles from the bulk-end of TZPs to the oolemma, or by the transit of EVs in the follicular fluid.

The importance of these novel communication mechanisms is exemplified by circumstances for which association with communication mediated by TZP or EVs has already been demonstrated, such as the accumulation of maternal transcripts in the oocyte, the acquisition of oocyte competence, and the decline in oocyte developmental potential associated with aging. Moreover, there is increasing evidence that assisted reproductive technologies disturbs the intrafollicular interactions, but their short and long-term effects are yet to be studied.

Studies in this field are limited, and in addition to the lack of knowledge about the effects of disrupting such communication mechanisms, there are plenty of unanswered questions in the subject area. Among other questions, it is unknown how the passage of molecules into the oocytes is regulated, and whether EVs in the follicular fluid can directly or indirectly modulate the oocyte. A thorough understanding of the biology of TZPs and EVs-mediated communication will potentiate the advancement of assisted reproductive technologies. These possibilities include modulation of the function of TZPs, regulation of EVs-cargo, and its use in *in vitro* culture conditions.
